# Enhancer heterogeneity in acute lymphoblastic leukemia drives differential gene expression in patients

**DOI:** 10.1182/blood.2024028019

**Published:** 2025-10-23

**Authors:** Alastair L. Smith, Nicholas Denny, Catherine Chahrour, Kim Sharp, Marta Arachi, Ana M. Dopico-Fernandez, Natalina Elliott, Joe R. Harman, Thomas Jackson, Huimin Geng, Owen Smith, Jonathan Bond, Irene Roberts, Ronald W. Stam, Nicholas T. Crump, James O. J. Davies, Anindita Roy, Thomas A. Milne

**Affiliations:** 1 Medical Research Council Molecular Haematology Unit, Weatherall Institute of Molecular Medicine, Radcliffe Department of Medicine, and University of Oxford, Oxford, United Kingdom; 2 Department of Paediatrics, University of Oxford, Oxford, United Kingdom; 3 Dark Blue Therapeutics Ltd, Oxford, United Kingdom; 4 Department of Laboratory Medicine, University of California, San Francisco, CA; 5 Trinity College, University of Dublin, Dublin, Ireland; 6 Systems Biology Ireland, School of Medicine, University College Dublin, Dublin, Ireland; 7 Children’s Health Ireland at Crumlin, Dublin, Ireland; 8 Princess Máxima Center for Pediatric Oncology, Utrecht, The Netherlands; 9 Hugh and Josseline Langmuir Centre for Myeloma Research, Centre for Haematology, Department of Immunology and Inflammation, Imperial College London, London, United Kingdom; 10 Radcliffe Department of Medicine, Oxford National Institute of Health Research Biomedical Research Centre, University of Oxford, Oxford, United Kingdom

## Abstract

Genetic alterations alone cannot account for the diverse phenotypes of cancer cells. Even cancers with the same driver mutation show significant transcriptional heterogeneity and varied responses to therapy. However, the mechanisms underpinning this heterogeneity remain underexplored. Here, we find that novel enhancer usage is a common feature in acute lymphoblastic leukemia (ALL). In particular, *KMT2A*::*AFF1* ALL, an aggressive leukemia with a poor prognosis and a low mutational burden, exhibits substantial transcriptional heterogeneity between individuals. Using single-cell multiome analysis and extensive chromatin profiling, we reveal that much transcriptional heterogeneity in *KMT2A*::*AFF1* ALL is driven by novel enhancer usage. By generating high-resolution Micro Capture-C data in primary patient samples, we identify patient-specific enhancer activity at key oncogenes such as *MEIS1* and *RUNX2*, driving high levels of expression of both oncogenes in a patient-specific manner. Overall, our data show that enhancer heterogeneity is highly prevalent in *KMT2A*::*AFF1* ALL and may be a mechanism that drives transcriptional heterogeneity in cancer more generally.

## Introduction

In higher eukaryotes, gene transcription initiates from promoters,^1^ but highly regulated context-specific expression requires the activity of distal regulatory elements, termed enhancers.^1,2^ Aberrant enhancer activity is being increasingly recognized as a driver of human disease.^3–7^ Key attributes of enhancer activity include a specific chromatin profile (enrichment for H3K27ac and H3K4me1); the production of short bidirectional transcripts termed enhancer RNAs; the binding of sequence-specific transcription factors (TFs); the tendency to come within close proximity of the promoter in 3-dimensional space when active; and the ability to drive tissue- and temporal-specific gene expression.^1,2,8–10^ Much work has focused on how large-scale genome rearrangements, DNA insertions, or single-nucleotide variants (SNVs) can alter TF binding and thus affect enhancer function, but less is known about how non-DNA mutations such as chromatin or epigenetic changes can affect enhancer function in cancer, or what impact this might have on transcriptional heterogeneity in patients.

Acute lymphoblastic leukemia (ALL) is the most common type of childhood cancer.^11,12^ Although children with ALL generally respond well to therapy, there are specific subtypes that still confer a poor prognosis,^12,13^ although new therapies such as blinatumomab have improved outcomes in infant ALL.^14^ Even so, there are limited treatment options for patients with ALL who relapse, ^15^ and even when successful, treatment of childhood ALL can have life-long adverse impacts.^16,17^

ALL with rearrangements of the *KMT2A* gene (formerly *MLL*) cause in-frame gene fusions creating novel fusion proteins.^18^ The most common *KMT2A* rearrangement is *KMT2A*::*AFF1*,^18^ which is the major cause of infant ALL and, relative to other subtypes, has a low mutational burden and no common cooperating mutations. ^13,19–23^ Despite the low mutational burden, *KMT2A* rearranged leukemias exhibit substantial transcriptional and phenotypic heterogeneity between individuals.^22,24^ KMT2A::AFF1 binds to the promoters of genes and drives aberrant activation through both transcriptional and epigenetic mechanisms.^25,26^ Recent work by us, and others, has shown that KMT2A::AFF1 binding to target genes can be influenced by the specific fusion breakpoint^27^ as well as the levels of the fusion protein itself.^28^ In addition, we have also shown that the KMT2A::AFF1 complex drives highly aberrant oncogenic enhancer activation.^29,30^ However, it is unknown how much enhancer usage might differ between patients with the same leukemia subtype, or how differential enhancer usage (as opposed to promoter binding) might drive individual transcription patterns and thereby influence prognostic outcomes.

To better understand the influence of enhancer usage in ALL, we first identified extensive transcriptional heterogeneity in *ETV6*::*RUNX1, DUX4*/*ERG*, and hyperdiploidy B-cell ALL (B-ALL) primary patient samples, and linked this to novel open chromatin regions that are most likely enhancers. Extensive chromatin profiling revealed the existence of many novel enhancers in individual patients with *KMT2A*::*AFF1*. CRISPR-Cas9–based deletion of representative enhancers, or degradation of components of the KMT2A::AFF1 transcription elongation complex, not only modified target gene expression in a cell-specific manner but also diminished enhancer-promoter interactions and other features of active enhancers. Notably, we identified patient-specific, KMT2A::AFF1-bound, enhancers near *MEIS1* and *RUNX2* and showed that expression of both genes was increased in this patient. To our knowledge, for the first time, we were able to use the high-resolution 3C technique Micro-Capture-C (MCC)^31^ in a primary patient sample to show, in detail, that these enhancers contact the *MEIS1* and *RUNX2* promoters, directly implicating these patient-specific enhancers in the overexpression of these genes. Taken together, our data suggest that enhancer heterogeneity is highly prevalent in primary ALL, and that this likely plays a significant role in the phenotypic diversity observed between individuals.

## Methods

### Patient samples

Infant (aged <1 year at diagnosis) and childhood (aged 1-18 years) ALL samples were obtained from Blood Cancer UK Childhood Leukaemia Cell Bank (now VIVO Biobank, United Kingdom) under their ethics approval (REC: 23/EM/0130), and from Our Lady’s Children’s Hospital, Crumlin, Ireland (REC: 21/LO/0195). Informed consent was obtained from all participants or those with parental responsibility.

### ATAC-seq/TOPmentation/CUT&Tag

Assay for transposase-accessible chromatin with sequencing (ATAC-seq) was conducted on 5 × 10^4^ live cells using Nextera Tn5 transposase (Illumina) as previously described.^29^ Libraries were sequenced by paired-end sequencing with a 75-cycle high-output Nextseq 500 kit (Illumina). TOPmentation was performed as described.^29^ Cleavage under targets and tagmentation (CUT&Tag) was performed as previously described.^32^ A brief protocol and data analysis details are provided in the supplemental Materials (available on the *Blood* website).

### Single-cell multiome

Cryopreserved bone marrow cells were thawed, and 1.6 × 10^4^ live CD19^+^ blasts of samples from 4 patients with *KMT2A-AFF1*^*+*^ ALL were sorted using a fluorescence-activated cell sorter and nuclei were extracted using the recommended protocol (Chromium). Male/female sample pairs were loaded together on 1 Chromium 10× lane for processing with the Single-Cell Multiome ATAC + Gene Expression protocol. Sequencing was performed on a NovaSeq 6000, with gene expression libraries sequenced on a PE150 S4 flow cell and ATAC-seq libraries on an SP PE50 flow cell. Data analysis details are provided in the supplemental Materials.

### RNA sequencing (RNA-seq) and quantitative reverse transcription–PCR

For quantitative polymerase chain reaction (PCR), RNA was extracted from 1 × 10^6^ cells with the RNeasy mini kit (Qiagen). Reverse transcription was conducted using Superscript III (Thermo Fisher Scientific) with random hexamer primers (Thermo Fisher Scientific), and complementary DNA was analyzed by TaqMan quantitative PCR, using the housekeeping gene *YWHAZ* for gene expression normalization (supplemental Table 1).

### MCC

MCC was performed as previously described,^33^ a brief protocol is provided in the supplemental Materials. Analysis was performed using the MCC pipeline.^31^

### Whole-genome sequencing

Genomic DNA was extracted from 5 × 10^6^ cells using a Monarch Genomic DNA extraction kit (NEB). Genomic DNA (2 ng) was incubated at 55°C for 15 minutes with 0.4 μL Illumina Tagment DNA Enzyme (Illumina) before purification (Qiagen MinElute PCR purification kit). Indexing and sample purification was performed in the same manner as for TOPmentation. Libraries were sequenced on a NovaSeq X (2 × 150 base pairs). Data analysis details are provided in the supplemental Materials.

## Results

### Heterogeneity of enhancer activity is common in B-ALL

We initially hypothesized that differential enhancer usage could be a major driver of heterogeneity in ALL. To explore this idea, we leveraged publicly available RNA-seq and ATAC-seq data sets from a diverse cohort of 24 primary patient samples^34^ comprised of *ETV6*::*RUNX1, DUX4*/*ERG*, and hyperdiploid B-ALL subtypes. Although each of the 3 subtypes clustered separately based on their transcriptional profile, hierarchical and optimized k-means clustering revealed 6 distinct gene expression clusters, underscoring within-subtype variability, even for a less diverse subtype such as *ETV6*::*RUNX1* (supplemental Figure 1A).

**Figure 1:**
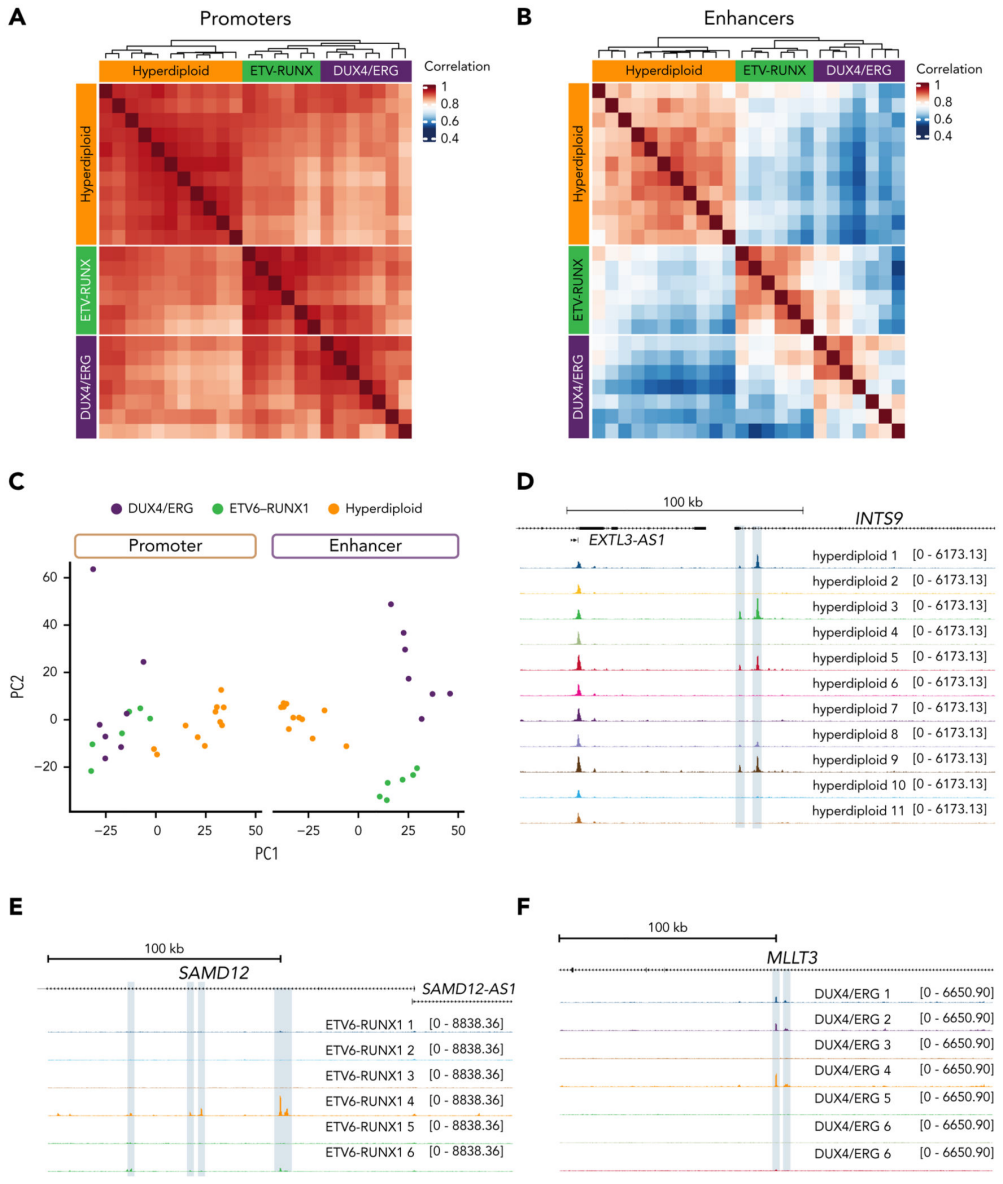
Patients with B-ALL display enhancer heterogeneity between individuals. (A) Correlation of accessibility at promoter regions (<2.5 kb from a TSS) measured by ATAC-seq signal between patient with *DUX4/ERG, ETV6-RUNX1* (ETV-RUNX), and hyperdiploid subtypes. Data obtained from GSE161501. (B) Correlation of accessibility at putative enhancers (≥2.5 kb from a TSS) as measured by ATAC-seq signal between *DUX4/ERG, ETV-RUNX*, and hyperdiploid subtypes. (C) Principal component analysis of chromatin accessibility at promoters (left) and enhancers (right) for all 3 B-ALL subgroups. (D) ATAC-seq at the *INTS9* locus for hyperdiploid samples. Putative enhancer regions with a high degree of intersample variability are highlighted in blue. (E) ATAC-seq at the *SAMD12* locus for *ETV6-RUNX1* samples. Putative enhancer regions with a high degree of intersample variability are highlighted in blue. (F) ATAC-seq at the *MLLT3* locus for *DUX4/ERG* samples. Putative enhancer regions with a high degree of intersample variability are highlighted in blue.

To investigate the source of this heterogeneity, we examined chromatin accessibility, generating a consensus peak set consisting of 71 800 open chromatin regions and categorized these into promoters (<2.5 kilobases [kb] from a transcription start site [TSS]) and putative enhancers (≥2.5 kb; potentially including nonenhancer loci). Promoter peaks exhibited a substantially higher correlation in accessibility (0.81-0.96; Figure 1A) between samples than enhancers (0.52-0.91; Figure 1B), supporting the proposition that variability in enhancer usage is a stronger signature of leukemia subtype. Moreover, enhancer regions were able to separate the B-ALL subtypes into distinct subsets, whereas promoter regions failed to fully distinguish the *ETV6*::*RUNX1* and *DUX4*/*ERG* subtypes (Figure 1C), consistent with enhancers being the main source of transcriptional differences.^35–37^

Next, we examined enhancer variability at the individual patient level. By intersecting the open chromatin regions from each sample with the consensus peak set (supplemental Figure 1B), we observed that enhancer regions displayed greater variability than promoter regions across all subtypes. Notable examples of loci with individual sample variable enhancer activity include *INTS9* in hyperdiploid B-ALL (Figure 1D), *SAMD12* in ETV6::RUNX1 (Figure 1E), and *MLLT3* in DUX4/ERG (Figure 1F). Taken together, these results show that, despite an enhancer activity signature shared by each B-ALL subtype, there is also significant variability between samples, suggesting patient-to-patient enhancer heterogeneity.

To evaluate whether enhancer activity differences influence gene expression, we compared scaled chromatin accessibility at putative enhancers with expression of the nearest gene. Despite the limitation that enhancers do not always regulate the adjacent promoter, we observed a notable positive correlation (r = 0.36-0.62) for the 100 most variable genes (supplemental Figure 1C). These findings support the idea that enhancer variability contributes to the transcriptional heterogeneity observed across patient samples.

### Altered enhancer activity regulates differential gene expression in *KMT2A*::*AFF1* ALL cell lines

**Figure 2:**
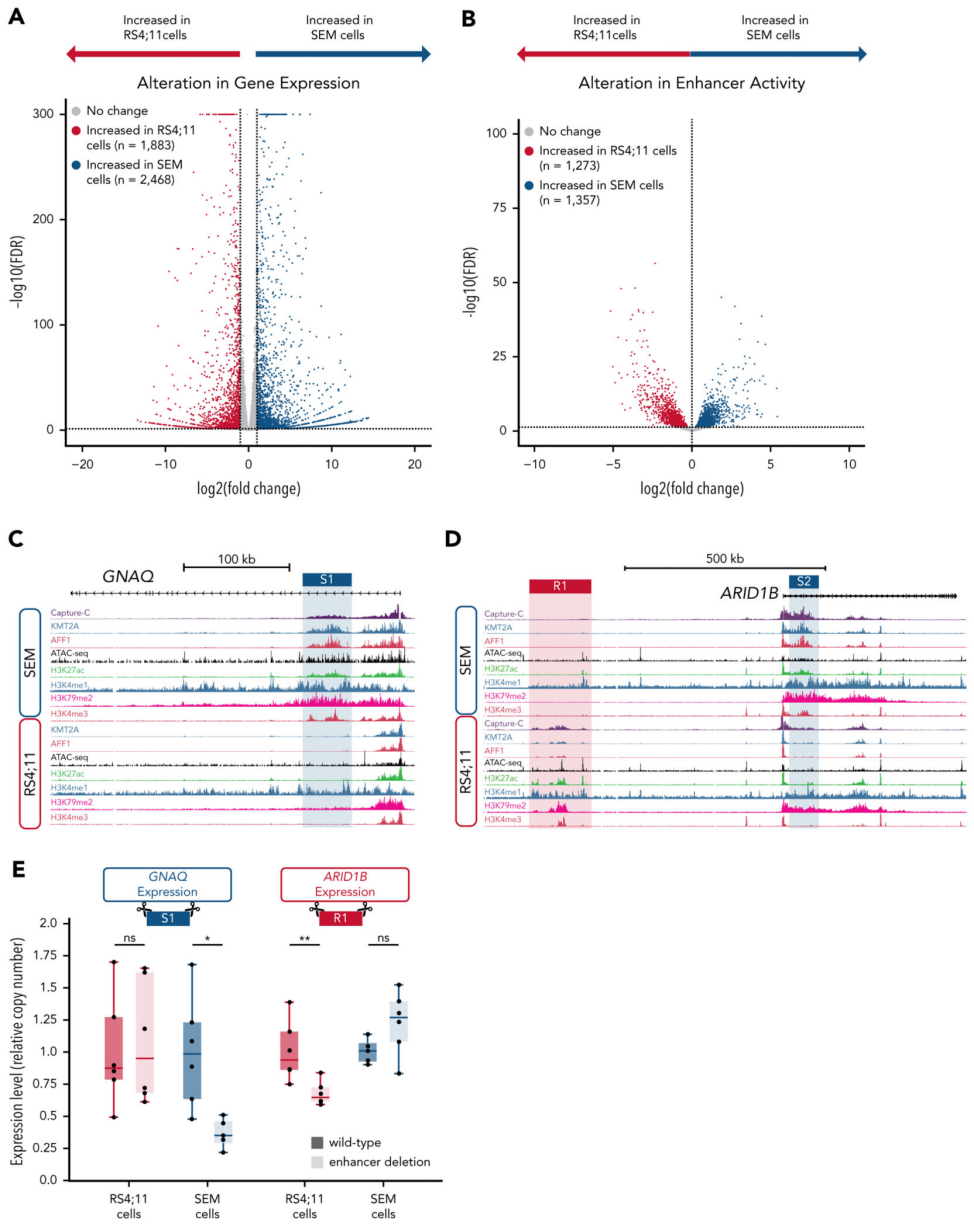
Differential enhancer regions in *KMT2A*::*AFF1* cell lines are functional enhancers that drive differential gene expression. (A) Volcano plot of differentially expressed genes between RS4;11 (1883; red) and SEM cells (2468; blue) or no significant change (gray) from 3 biological replicates; false discover rate (FDR) of <0.05. (B) Volcano plot of enhancers with significantly increased accessibility in RS4;11 (1273; red) or SEM cells (1357; blue), or enhancers with unaltered accessibility (gray) from 8 biological replicates, FDR < 0.05. (C) Chromatin immunoprecipitation sequencing (ChIP-seq) tracks at the *GNAQ* locus for KMT2A, AFF1, H3K27ac, H3K4me1, H3K79me2, and H3K4me3 together with ATAC-seq and Capture-C in SEM cells using the *GNAQ* promoter as a viewpoint. The SEM-specific *GNAQ* enhancer (S1) is highlighted in blue. (D) ChIP-seq tracks at the *ARID1B* locus for KMT2A, AFF1, H3K27ac, H3K4me1, H3K79me2, and H3K4me3 together with ATAC-seq and Capture-C in SEM cells using the *ARID1B* promoter as a viewpoint. The RS4;11 specific intergenic enhancer (R1) is highlighted in red, and the SEM-specific intragenic enhancer (S2) is highlighted in blue. (E) Reverse transcription-qPCR comparing the expression of enhancer deletion mutants (light shading) with wild type (dark shading) in RS4;11 (red) or SEM cells (blue) when deleting either the intragenic GNAQ enhancer (S1; left) or ARID1B intergenic enhancer (R1; right). Significance of alterations in relative copy number were determined by a 2-sided *t* test with correction for multiple testing (Benjamini-Hochberg), n = 6 biological replicates. *Adjusted *P* value < .05; **P < .01. ns, not significant.

To explore the mechanistic aspects of differential enhancer activity in greater detail, we focused on *KMT2A*::*AFF1* ALL. We first analyzed the patient-derived cell lines SEM and RS4;11. RNA-seq of these cell lines revealed 4351 differentially expressed genes (Figure 2A), indicating significant transcriptional heterogeneity between them.

To compare enhancer usage between the 2 cell lines, we intersected regions of chromatin accessibility of ≥2.5 kb from a TSS with regions of enriched H3K27ac, identifying 7194 putative enhancers. We removed 989 regions with potential copy number variation (supplemental Table 3) and identified 1357 enhancers that displayed significantly increased activity in SEM cells, with 1273 increased in RS4;11 cells (Figure 2B; supplemental Figure 2A) with a substantial number of enhancers being uniquely detected in one of these cell lines.

Strikingly, we identified enhancers with differential activity at known KMT2A::AFF1 target genes such as *GNAQ* and *ARID1B*.^29^ The *GNAQ* gene is associated with intragenic enhancers in SEM cells, which are absent in RS4;11 cells (Figure 2C, blue shading). In contrast, at *ARID1B*, an upstream intergenic enhancer is present in RS4;11 cells but absent in SEM (Figure 2D, red shading), and an intragenic enhancer in SEM cells shows reduced activity in RS4;11 (Figure 2D, blue shading). At both of these genes, the cell line–specific enhancers show an increased frequency of interaction with the promoter, as measured by Capture-C (Figure 2C-D).^10^

We validated the activity of the differential *GNAQ* and *ARID1B* enhancers by targeting them for CRISPR-Cas9–mediated deletion (supplemental Figure 2B). Deletion of the *GNAQ* intragenic enhancer locus decreased expression of *GNAQ* in SEM cells, whereas in RS4;11 cells no significant change in *GNAQ* expression was observed (Figure 2E). Similarly, deletion of the RS4;11-specific *ARID1B* enhancer sequence in RS4;11 cells resulted in a reduction in gene expression, whereas RNA levels were not significantly altered in SEM deletion mutants (Figure 2E). This is consistent with these regions being active enhancers in a cell line–specific manner.

To explore the effect of cell line–specific enhancers on the transcription genome-wide, we linked enhancers to the nearest gene. Enhancers displaying increased activity were more frequently linked to genes displaying increased expression (51.8% of enhancer-gene pairs in SEM cells, and 64.9% in RS4;11 cells; supplemental Figure 2C). In addition, genes linked to ≥1 differentially active enhancer(s) displayed significantly increased differences in gene expression (supplemental Figure 2D). Thus, these regions are bona fide enhancers with differential effects on gene expression.

### Novel enhancer activity can arise from the same donor cells in a *KMT2A*::*AFF1* primary B-ALL model

One of the limitations of cell line models such as RS4;11 and SEM is that they harbor a number of additional potentially pathogenic mutations other than the *KMT2A*::*AFF1* translocation (supplemental Figure 3A; supplemental Tables 3 and 4). To determine whether enhancer heterogeneity is an intrinsic feature of KMT2A::AFF1 leukemia or simply a reflection of the mutational differences between these cell lines, we wanted to use a genetically uniform system for deriving a KMT2A::AFF1 leukemia.

**Figure 3:**
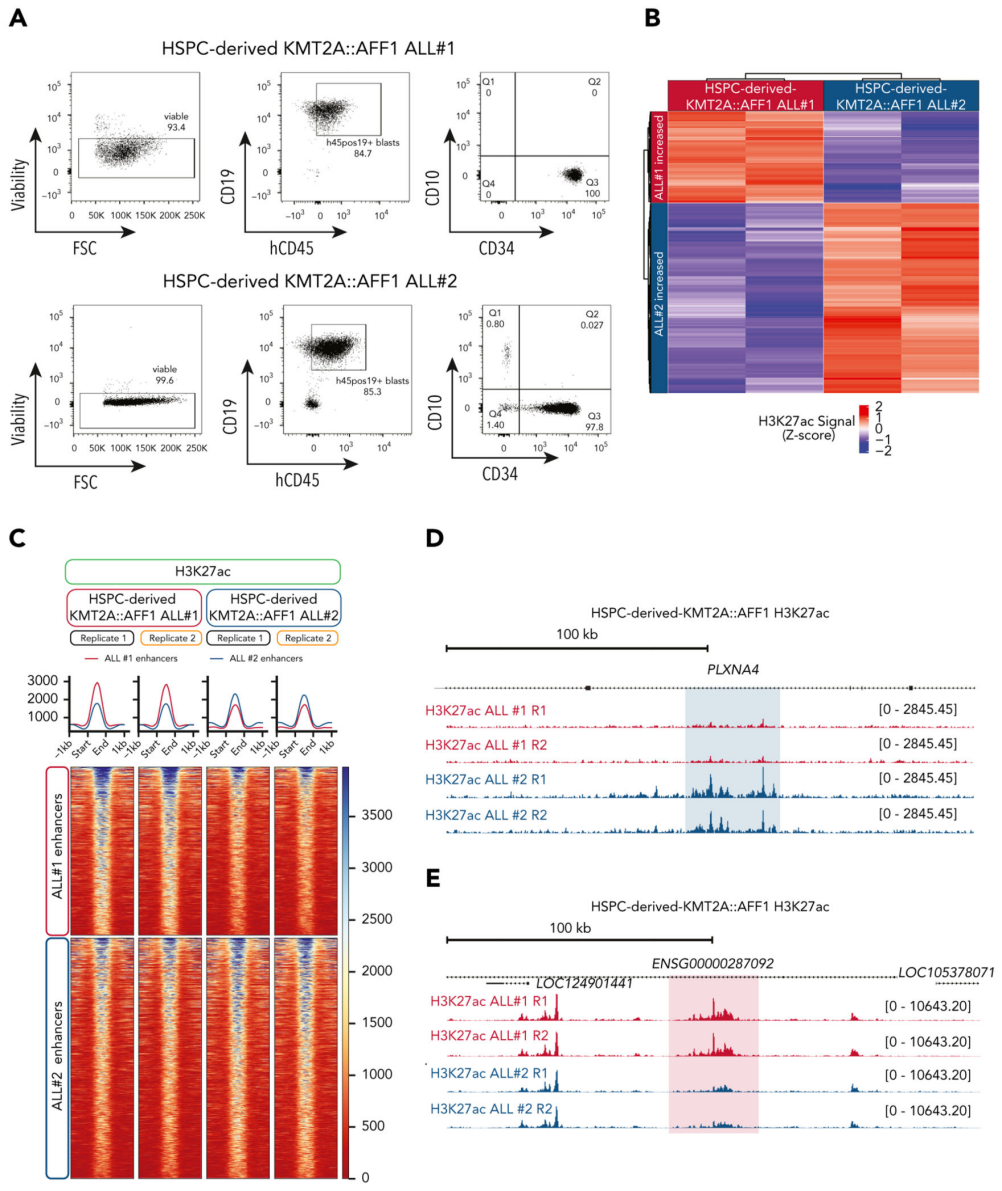
Enhancer heterogeneity persists in genetically matched KMT2A::AFF1 leukemias derived from a single donor. (A) Representative flow cytometry plots of the sorting strategy used for KMT2A::AFF1 ALL samples. (B) Scaled H3K27ac level at the 500 most variable enhancer peaks between the 2 KMT2A::AFF1 models. (C) Tornado plot of H3K27ac CUT&Tag signal in 2 HSPC-derived KMT2A::AFF1 ALL models at the 500 most variable enhancer peaks, k-means clustering separates the regions into enhancers showing increased activity in KMT2A::AFF1 ALL 1 (top) vs KMT2A::AFF1 ALL 2 (bottom). (D) CUT&Tag for H3K27ac at the *PLXNA4* locus, enhancer regions with increased activity in KMT2A::AFF1 ALL 1 are highlighted in blue. (E) CUT&Tag for H3K27ac at the *ENSG00000287092* locus, enhancer regions with increased activity in KMT2A::AFF1 ALL 2 are highlighted in red. FSC, forward scatter; HSPC, hematopoietic stem and progenitor cell.

Using our previously published model in which we are able to create a KMT2A::AFF1 leukemia de novo using CRISPR editing in human fetal hematopoietic stem and progenitor cells,^38^ we analyzed 2 leukemic samples derived from the same biological donor to determine whether they would still exhibit divergent enhancer landscapes. CD19^+^ leukemic blasts were isolated from the bone marrow of 2 tertiary KMT2A::AFF1 xenograft models (Figure 3A) and we used CUT&Tag for H3K27ac to identify enhancer regions. Although these tertiary ALLs displayed high similarity both immunophenotypically (Figure 3A) and in regulatory element activity (supplemental Figure 3B-C), we identified striking differences in enhancer usage (Figure 3B-C). In particular, we observed discrete enhancer activity patterns at several loci (Figure 3D-E). These findings highlight the persistence of enhancer-level heterogeneity even in leukemic cells derived from the same donor population.

### Novel enhancers exist in samples from patients with *KMT2A*::*AFF1* B-ALL leukemia

**Figure 4:**
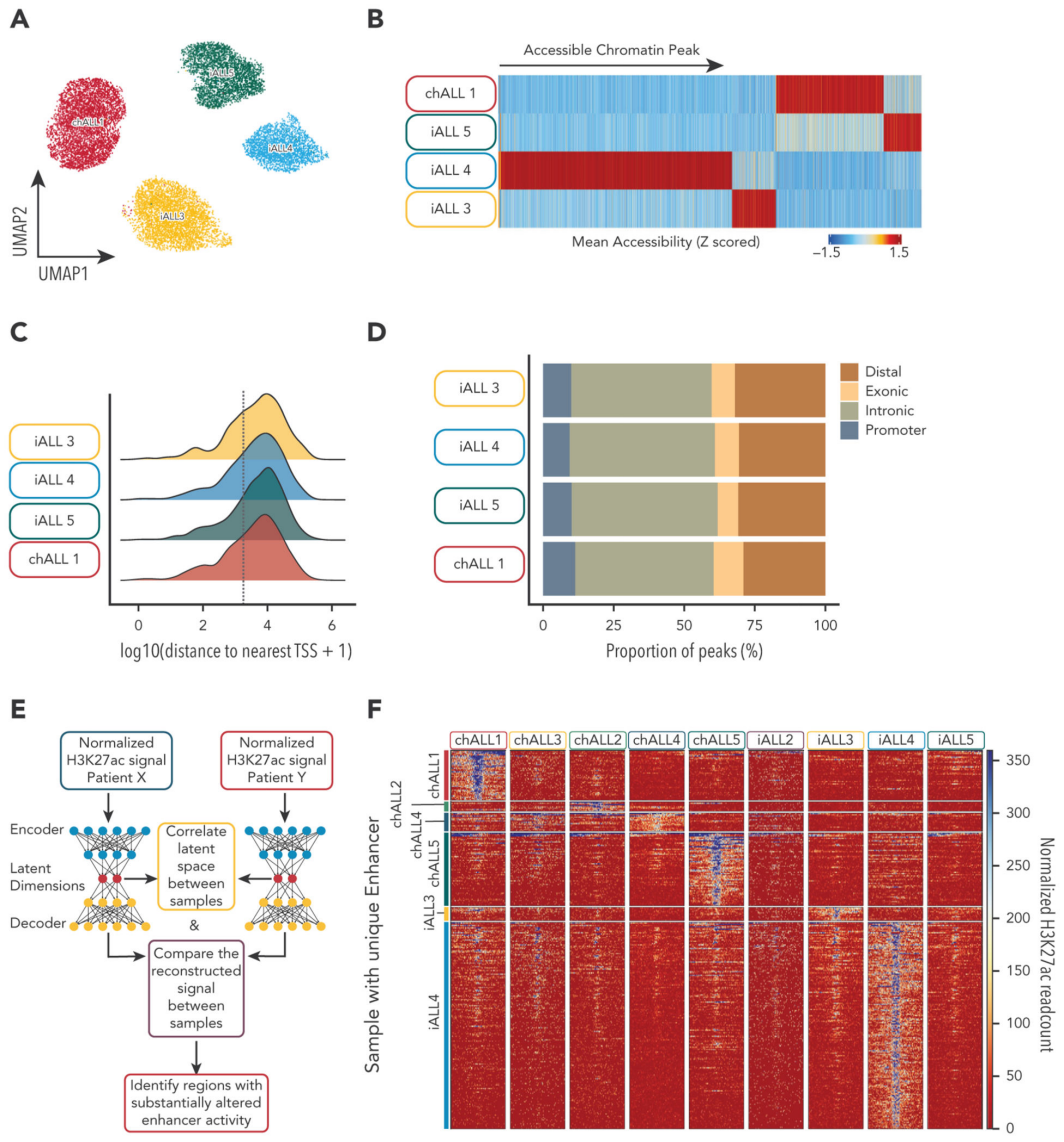
Differential enhancer regions in *KMT2A*::*AFF1* patients are readily observed. (A) UMAP of the single-cell ATAC-seq modality for 4 KMT2A::AFF1 blast samples from the VIVO Biobank, United Kingdom. (B) Regions of accessible chromatin displaying significantly increased accessibility in 1 of 4 patient samples. (C) Genomic distribution of uniquely accessible ATAC-seq peaks relative to the nearest TSS, the dotted gray line indicates 2.5 kb. (D) Annotation of the genomic location of unique ATAC-seq peaks. (E) Schematic of the strategy used to identify unique enhancer peaks using H3K27ac ChIP-seq data sets. (F) Tornado plot of H3K27ac signal in KMT2A::AFF1 samples at enhancers identified as being patient specific. UMAP, uniform manifold approximation and projection.

Next, we sought to examine how prevalent heterogeneity in enhancer activity is in patient samples. To directly compare enhancer usage and gene expression on a single-cell level, we performed 10× Genomics single-cell Multiome (ATAC-seq + RNA-seq) on fluorescence-activated cell sorted CD19^+^ blast populations obtained from 4 samples from patients with *KMT2A*::*AFF1* ALL (3 infants and 1 older child). Using the single-cell ATAC modality (Figure 4A), we examined the degree of heterogeneity in chromatin accessibility and identified 6231 regions of open chromatin with significantly altered accessibility between leukemic blasts from the 4 patients (340 494 regions identified in total; Figure 4B). Most (64%) differentially accessible regions were located >2.5 kb from the nearest TSS (Figure 4C), predominantly consisting of intronic elements (Figure 4D), implying that most of these regions are putative enhancers.

To robustly identify active enhancers, we performed detailed epigenetic profiling in 9 *KMT2A*::*AFF1* patient samples (4 infants and 5 children) with a low cell number–optimized chromatin immunoprecipitation protocol, TOPmentation^29^ (supplemental Table 5). We defined a consensus set of putative enhancer regions by identifying H3K27ac-enriched regions that were ≥2.5 kb from the nearest TSS. Because of the limited material available, resulting in a lack of replicates and variability in sample viability, we devised a deep learning–based strategy to limit sample-to-sample noise and identify enhancer regions that displayed substantially altered enhancer activity, marked by increased levels of H3K27ac, between patient blasts (Figure 4E). This allowed us to identify 290 patient-specific putative enhancers across the 9 patient samples (Figure 4F; supplemental Figure 4A-F). In many cases, these patient-specific enhancers were associated with the binding of KMT2A (supplemental Figure 4A-F).

**Figure 5:**
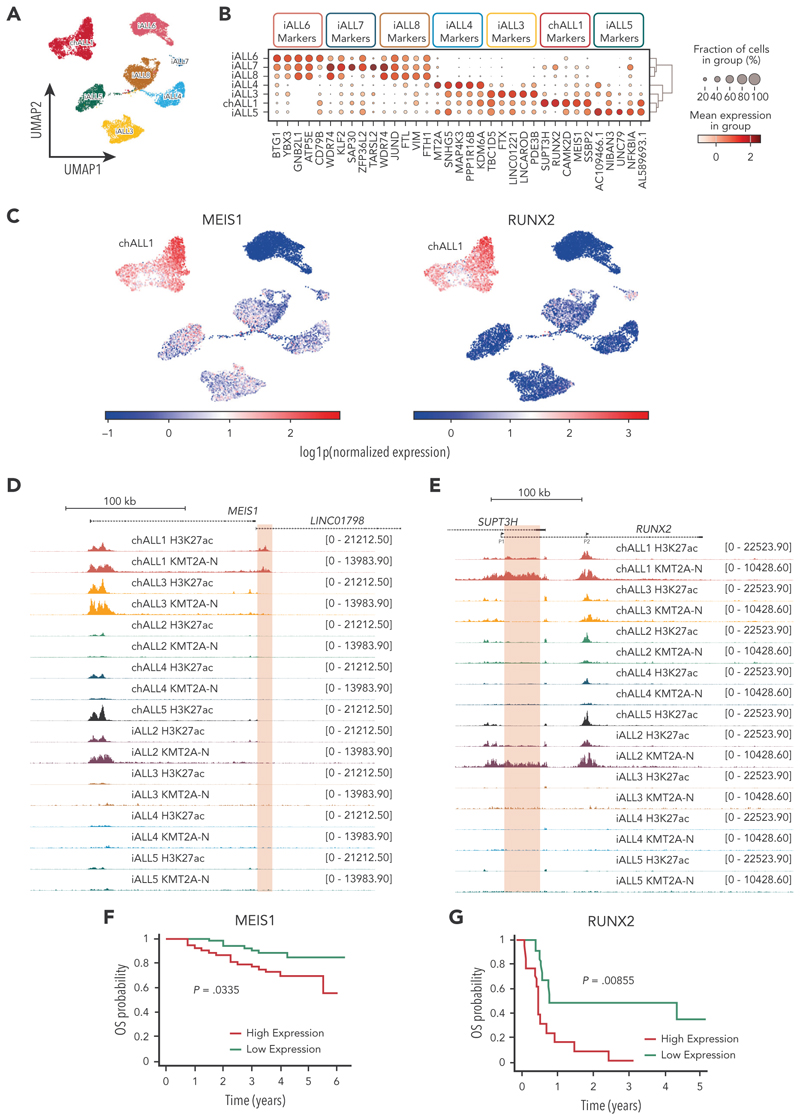
Differential enhancer activity in *KMT2A*::*AFF1* patients drives oncogene specific expression such as at *MEIS1* and *RUNX2*. (A) UMAP of single-nucleus gene expression (snGEX) for 4 KMT2A::AFF1 blast samples (chALL1, iALL3-5; VIVO Biobank, United Kingdom) and 3 single-cell GEX (scGEX) samples (EGAS00001003986). (B) Dot plot of marker gene analysis between 7 KMT2A::AFF1 blast samples, showing the top 5 marker genes per sample. (C) Normalized *MEIS1* (left) and *RUNX2* (right) expression in KM2TA::AFF1 sn/scGEX samples. (D) TOPmentation for H3K27ac and the N terminus of KMT2A (KMT2A-N) in KMT2A::AFF1 blast sample (VIVO Biobank, United Kingdom) at the *MEIS1* locus. The chALL1 unique enhancer region downstream of *MEIS1* is highlighted in red. (E) TOPmentation for H3K27ac and KMT2A-N in KMT2A::AFF1 blast samples at the *RUNX2* locus. The chALL1- and iALL2-specific enhancer region upstream of *RUNX2* is highlighted in red. (F) Survival curve comparing high (red) and low (green) *MEIS1* expression in B-ALL. Data analyzed from COG P9906 childhood B-ALL clinical trial. (G) Survival curve comparing high (red) and low (green) *RUNX2* expression in B-ALL. Data analyzed from the Eastern Cooperative Oncology Group E2993 adult B-ALL clinical trial. UMAP, uniform manifold approximation and projection.

Because we observed patient-specific enhancer activity, we sought to determine the extent of transcriptional heterogeneity between *KMT2A*::*AFF1* samples. To this end, we integrated our single-nucleus gene expression data with a published single-cell gene expressions^24^ data set of 3 *KMT2A*::*AFF1* infant ALL blasts to increase the number of patients in our data set. Initial dimensionality reduction, after batch correction (Figure 5A), indicated that each patient sample formed a distinct cluster, implying unique gene expression profiles. Moreover, analysis of differential gene expression between the samples (Figure 5B; false discovery rate of <0.01) revealed a surprising number of genes (3306) that exhibited heterogeneous expression. This number is comparable with the differential expression observed between *KMT2A*::*AFF1* cell lines (Figure 2A).

Interestingly, marker gene analysis indicated that 2 key *KMT2A*::*AFF1* target genes, *MEIS1* and *RUNX2*, were both highly elevated in chALL1 (Figure 5B-C; supplemental Figure 5A). Returning to our epigenetic data from this patient, we observed a chALL1 unique putative enhancer region downstream of *MEIS1* (Figure 5D) and a putative enhancer upstream of *RUNX2* in chALL1 and iALL2 (Figure 5E), both of which were enriched for KMT2A binding. Increased expression of either *MEIS1* or *RUNX2* is associated with a significantly lower overall survival probability (Figure 5F-G), and also correlates with either relapse (supplemental Figure 5B-C) or decreased remission (supplemental Figure 5D). Together, these results show that high levels of expression of 2 key genes associated with a worse prognosis (ie, *RUNX2* and *MEIS1*) are associated with nearby enhancer activity that is specific to an individual leukemia.

### MCC reveals that novel enhancers in *KMT2A*::*AFF1* samples directly regulate nearby putative target genes

**Figure 6:**
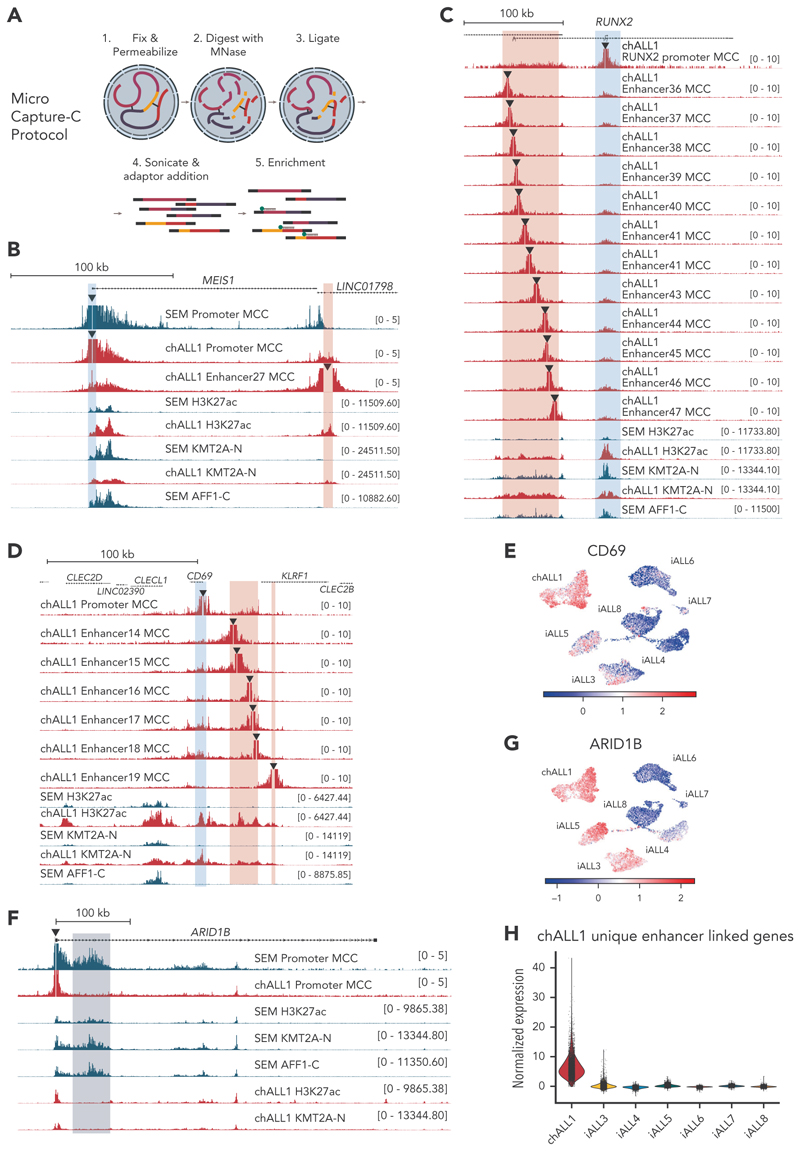
MCC reveals patient-specific enhancer-promoter interactions in primary patient cells. (A) A schematic for the MCC protocol. (B) MCC at the *MEIS1* locus using the promoter (blue highlight) as the viewpoint (triangle) for SEM cells (blue) or chALL1 cells (red), or from the chALL1-unique enhancer region (enhancer 27; red highlight), together with TOPmentation for H3K27ac and KMT2A-N. (C) MCC at the *RUNX2* locus in chALL1 cells using either the promoter (blue highlight) or open chromatin regions (enhancers 36-47) within the identified enhancer region (red highlight) as the viewpoint (triangle) together with TOPmentation for H3K27ac and KMT2A-N in SEM and chALL1 cells. (D) MCC at the *CD69* locus in chALL1 cells using either the promoter (blue highlight) or open chromatin regions (enhancers 14-19) within the identified enhancer regions (red highlight) as the viewpoint (triangle) together with TOPmentation for H3K27ac and KMT2A-N in SEM and chALL1 cells. (E) Expression of *CD69* in patients-derived KMT2A::AFF1 blast samples. (F) MCC for the *ARID1B* viewpoint (triangle) together with ChIP-seq for H3K27ac and KMT2A-N at the *ARID1B* locus. The SEM-specific intragenic enhancer is highlighted in blue. (G) Expression of *ARID1B* in patient-derived KMT2A::AFF1 blast samples. (H) Expression of genes linked to chALL1-unique enhancer regions between KMT2A::AFF1 single-cell blast samples.

To confirm that the putative enhancers identified in the chALL1 sample were bona fide enhancers, we performed the high-resolution 3C method, MCC^31^ (Figure 6A). MCC in cells of patients with chALL1 revealed direct interactions between the putative enhancers and the promoters of *MEIS1, RUNX2*, and *CD69* (Figure 6A-D; supplemental Figure 6C). Importantly, in SEM cells, MCC revealed an interaction between the *MEIS1* promoter and the enhancer within the *LINC01796* locus, compatible with the absence of the *MEIS1* 3^′^ enhancer in SEM cells (Figure 6B). Consistent with the presence of novel enhancers in chALL1, *CD69* also displayed higher expression levels in chALL1 cells (Figure 6E; supplemental Figure 6A). Interestingly, patient-specific enhancers have previously been identified at the *CD69* gene in cases of mixed-phenotype acute leukemia.^39^ Taken together, our data confirm that the differential enhancer regions identified at *MEIS1, RUNX2*, and *CD69* are indeed enhancers for these genes.

In contrast to *MEIS1, RUNX2*, and *CD69, ARID1B* expression was not substantially increased in chALL1 when compared with other patient samples, likely because an *ARID1B* intragenic enhancer that is present in SEM cells is missing in chALL1 (Figures 2D and 6F-G; supplemental Figure 6B,D).

Using MCC to link multiple chALL1-specific enhancers to specific genes, we observed a significant increase in the expression of target genes in chALL1 compared with all other patient blasts (Figure 6H; supplemental Figure 6E), implicating novel enhancer usage in oncogene overexpression.

### KMT2A::AFF1 binding is predictive of enhancer activity

**Figure 7:**
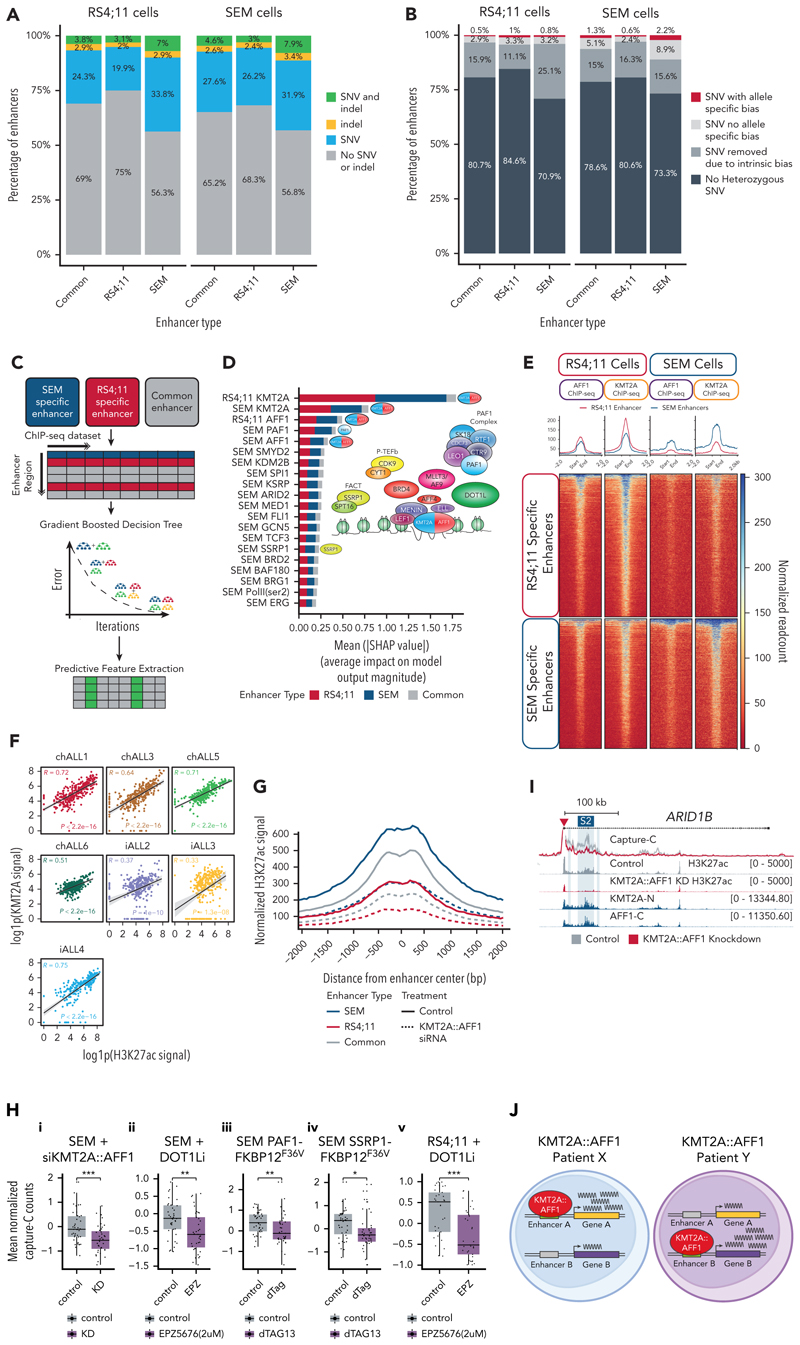
An unbiased machine-learning model identifies KMT2A::AFF1 complex binding as a driver of differential enhancer usage. (A) Proportion of enhancers (common = no change in activity, RS4;11 = increased activity in RS4;11 cells, SEM = increased activity in SEM cells) containing either an SNV (blue), an indel (yellow), or both (green) in RS4;11 cells (left) or SEM cells (right). (B) Proportion of enhancers of each enhancer type (common, RS4;11, SEM) containing no heterozygous SNVs (dark gray), SNVs removed due to intrinsic bias (eg, problematic genomic regions or mapping bias; gray), SNVs without allele-specific bias in accessibility (light gray), or those exhibiting allele specific bias in accessibility as measured by ATAC-seq (red). (C) Schematic of the strategy used to determine key predictive features of differential enhancer activity. ChIP-seq signal for 56 factors was extracted over enhancers with increased activity in RS4;11 cells (red; 1522), or SEM cells (blue; 1677) or those common to both (gray; 4232). A gradient boosted decision tree was trained from these data, and predictive features were extracted using SHAP. (D) The relative feature importance for each enhancer category (increased activity in SEM cells [blue], RS4;11 cells [red], or common enhancers [gray]) of the top 20 most important features for differential enhancer prediction. Features that correspond to binding of the KMT2A::AFF1 complex are highlighted, and a schematic of the complex is provided for reference. (E) Tornado plot of AFF1-C and KMT2A-N ChIP-seq signal at enhancers displaying increased activity in RS4;11 cells (top) or SEM cells (bottom) in RS4;11 (left) or SEM (right) cells. (F) Pearson correlation between H3K27ac and KMT2A signal at the 290 blast-specific enhancers identified, for each patient sample. (G) H3K27ac ChIP-seq signal at enhancers with increased activity in SEM cells (blue), RS4;11 cells (red), or common enhancers (gray) upon KMT2A::AFF1 knockdown by small-interfering RNA (siRNA; dashed line). (H) Enhancer-promoter interaction frequency at enhancer regions with increased activity in SEM cells (i-iv) or RS4;11 cells (v) upon treatment of SEM (i) or RS4;11 (v) cells with 2 μM EPZ5676 for 1 week or SEM PAF1-FKBP12^F36V^ (iii)/SEM SSRP1-FKBP12^F36V^ (iv) cells treated with dTag13 for 24 hours, together with SEM cells treated with an siRNA against KMT2A::AFF1 (i). Interaction frequency for each enhancer-promoter pair is shown relative to the mean interaction frequency of the control; n = 3 biological replicates per condition. *P < .05; **P < .01; ***P < .001. (I) Example of a loss of enhancer-promoter interactions at the *ARID1B* locus in SEM cells as assessed by Capture-C in control (gray) or KMT2A::AFF1 knockdown conditions (red) in 3 biological replicates. Enhancers with increased activity in SEM cells are highlighted in blue. ChIP-seq for H3K27ac in control (gray) or KMT2A::AFF1 knockdown conditions (red) in addition to the N terminus of KMT2A and the C terminus of AFF1 are provided for reference. (J) Model for the role of the KMT2A::AFF1 complex in promoting transcription heterogeneity between patients. bp, base pair; dTag, dTAG-13; EPZ, EPZ5676; indel, insertion-deletion; KD, knockdown; SHAP, SHapley Additive exPlanations.

Having identified differential enhancer usage in KMT2A::AFF1 leukemias, we wanted to explore what might be causing these differences. First, we looked for the presence of small-scale changes in DNA sequence at these enhancers. Using whole-genome sequencing we identified SNVs within the enhancers exhibiting differential activity in the KMT2A::AFF1 cell lines (SEM and RS4;11). Only 25% to 43.2% of enhancers contained at least 1 single-nucleotide variant (SNV) or insertion-deletion, and frequencies were comparable between common and cell line–unique enhancers (Figure 7A). Furthermore, we examined whether any of these variants exhibited allele-specific bias in our ATAC-seq data, because increased chromatin accessibility might indicate a functional consequence of the mutation. Strikingly, only 0.5% to 2.2% of enhancers contained ≥1 heterozygous SNVs exhibiting allele-specific bias (Figure 7B). Although we cannot exclude the possibility that these mutations have functional consequences through mechanisms such as disruption of topologically associated domain boundaries, taken together, these data suggest that SNVs in these enhancer regions are unlikely to be the major cause of differential enhancer activity between the cell lines.

Our past work has shown that KMT2A::AFF1 binding is essential for maintaining enhancer function at a shared set of KMT2A::AFF1 target genes.^29^ The unique enhancers identified in this current study often have KMT2A bound to them, implicating KMT2A::AFF1 in driving enhancer heterogeneity. To explore this in an unbiased manner, we created a model to identify the most likely factors driving enhancer heterogeneity in KMT2A::AFF1 ALL. We developed a gradient-boosted decision-tree model using 56 laboratory-generated chromatin immunoprecipitation–sequencing data sets from SEM and RS4;11 cells (Figure 7C; supplemental Table 6) that was able to predict which enhancers exhibit increased activity in SEM or RS4;11 cells (weighted F1 score of 0.845 for predicting enhancers increased in RS4;11 and 0.736 SEM specific enhancers; supplemental Figure 7A-B), as well as those with unchanged activity. We next used this model to identify the data sets with the highest predictive power (Figure 7D) and verified that the 5 most predictive features displayed significant enrichment differences between common and cell type–specific enhancer classes (supplemental Figure 7C).

Strikingly, the model identified the presence of KMT2A in each cell line as the most important feature for defining differential enhancer usage (Figure 7D) followed by its fusion partner AFF1 and the KMT2A::AFF1 complex component PAF1.^29,40,41^ Taken together, this implicates binding of the KMT2A::AFF1 complex in differential enhancer usage. In line with this, KMT2A and AFF1 chromatin immunoprecipitation–sequencing signal in both cell lines was enriched at cell-specific active enhancers and depleted at inactive enhancers (Figure 7E). This is also consistent with our observation of KMT2A binding at patient-specific enhancers (supplemental Figure 4A-F). We examined this more formally and observed a positive correlation between H3K27ac signal intensity and KMT2A binding at the unique enhancers in each patient (R = 0.33-0.75; Figure 7F) in further support of our model predictions. Furthermore, we also observed the presence of FLAG-KMT2A::Aff1 at these enhancer regions in a model derived from CD34^+^ cord blood,^42^ implying that it is the fusion protein present at these regions as opposed to the presence of wild-type KMT2A or AFF1 (supplemental Figure 7D).

We sought to establish a causative role for KMT2A::AFF1 at these differential enhancers by examining the effect of perturbations of the KMT2A::AFF1 complex in SEM cells. Knockdowns of KMT2A::AFF1^29^ reduced binding to SEM-specific enhancers (supplemental Figure 7E), as well as H3K27ac levels, indicating a reduction in enhancer activity (Figure 7G). In an analysis of previous Capture-C data, we also observed a significant decrease in enhancer-promoter interaction frequency for SEM-specific enhancers upon KMT2A::AFF1 knockdown^29^ (Figure 7Hi,I; supplemental Figure 7F), inhibition of DOT1L^43^ (EPZ5676; Figure 7Hii), or degradation of either PAF1-FKBP12^F36V^ (Figure 7Hiii) or SSRP1-FKBP12^F36V^ (Figure 7Hiv) via dTAG-13 treatment.^29^ We also observed a reduction of enhancer-promoter interactions at RS4;11-specific enhancers upon DOT1L inhibition in RS4;11 cells^43^ (Figure 7Hv).

Taken together, these data implicate the binding of KMT2A::AFF1 in the activity of these cell line–specific and patient-specific enhancers, suggesting that differential binding of the KMT2A::AFF1 complex is a key driver of transcriptional heterogeneity due to the regulation of differential enhancer activity (Figure 7J).

## Discussion

Much work has gone into developing targeted therapies, but even cancers with the same driver mutations show varied responses to therapy, along with significant transcriptional heterogeneity.^24,44–46^ Here, we have shown that enhancer heterogeneity is a common feature of *KMT2A*::*AFF1* ALL, and could be a major driver of transcriptional heterogeneity between patients. When important oncogenes such as *MEIS1* and *RUNX2* are overexpressed because of novel enhancer usage, this has clear implications for therapeutic response and patient outcomes in leukemia. Variability in enhancer activity has been observed across various cancers, including gastric adenocarcinoma,^47^ prostate tumors,^48^ and luminal breast cancer,^49,50^ suggesting that this may represent a broader mechanism contributing to heterogeneity across cancer types.

A key question arising from our findings is the origin of these novel enhancers. One hypothesis is that patient-specific SNVs may drive differential enhancer activity by altering TF binding sites, a mechanism proposed for donor-derived lymphoblastoid lines.^51^ However, our analysis of sequencing data from the SEM and RS4;11 cell lines revealed only a small number of SNVs associated with novel enhancers, despite the extensive epigenetic differences observed. This suggests that genetic variation at these enhancers alone is unlikely to fully account for the emergence of patient-specific enhancers in this context. We identify the differential binding of the *KMT2A*::*AFF1* fusion protein as a potential driver of differential enhancer usage, and one possibility is that mutations in other proteins (such as NSD2 or KMT2C, as observed in the SEM and RS4;11 cell lines) may play a role in influencing KMT2A::AFF1 binding profiles.

Recent work has suggested that the expression level of the KMT2A::AFF1 fusion protein is a predictor of lymphoid vs myeloid gene expression signatures due to differential fusion protein binding to target regions.^28^ Furthermore, the location of the KMT2A::AFF1 breakpoint has also been shown to influence the binding of the fusion protein and gene expression profiles.^27^ Taken together, one possible mechanism by which differential enhancer activity may emerge is due to the differential expression and binding of the fusion protein, dependent on the fusion breakpoint location. However, we did not observe differences in KMT2A::AFF1 expression between SEM or RS4;11 cells (supplemental Figure 7G), suggesting that variation in fusion protein levels is unlikely to explain the observed differences in enhancer activity.

Another possibility is that the leukemia initiating cell may provide a unique and distinct epigenetic landscape for KMT2A::AFF1 binding, driving differential enhancer usage. Indeed, because the SEM cell line was established from the peripheral blood of a 5-year-old girl at relapse,^52^ whereas RS4;11 cells were established from the bone marrow of a 32-year-old woman with ALL at relapse,^53^ some of the chromatin-associated and transcriptomic differences observed in these lines may reflect differences in the cell of origin of the leukemia. However, because each of several patients with KMT2A::AFF1 ALL displayed a distinct pattern of enhancer usage, and we observed differential enhancer usage in our hematopoietic stem and progenitor cell–derived model, cell type is unlikely to fully explain the effects observed. It is possible that a stochastic element contributes to initial enhancer activation and is subsequently reinforced by stabilization of KMT2A::AFF1 binding. For example, KMT2A::AFF1 binding has been shown to be altered by DNA methylation,^54,55^ so stochastic and developmental stage–specific differences in DNA methylation patterns in progenitor cells could be a driver of differential binding and enhancer usage.

It is likely that multiple factors control the emergence of differential enhancer usage, but our work here establishes variable enhancer usage as a common driver of transcriptional heterogeneity in multiple leukemias, suggesting this could be a common mechanism underpinning an important aspect of patient heterogeneity.

## Key Points

- Differential enhancer activity contributes to oncogene overexpression and transcriptional diversity in primary KMT2A::AFF1 leukemia samples.
- Individual leukemias can develop unique enhancer profiles even when initiating from the same starting material.
